# A Rare Complication of Gastric Volvulus in Infectious Mononucleosis: A Case Report

**DOI:** 10.7759/cureus.103230

**Published:** 2026-02-08

**Authors:** Xiaoqing Wu, Ling Deng, Yan Chen, Xiaoling Zhou, Jingrong Yang, Yue Song

**Affiliations:** 1 Department of Pediatrics, Xindu District People's Hospital, Chengdu, CHN; 2 Department of Radiology, Xindu District People's Hospital, Chengdu, CHN; 3 Department of Radiology, Chengdu Women's and Children's Central Hospital, School of Medicine, University of Electronic Science and Technology of China, Chengdu, CHN; 4 Department of Pediatrics, First Affiliated Hospital of Chengdu Medical College, Chengdu, CHN

**Keywords:** children, complication, gastric volvulus, infectious mononucleosis (im), splenomegaly

## Abstract

Infectious mononucleosis (IM), typically resulting from Epstein-Barr virus infection, is characterized by the clinical triad of fever, pharyngitis, lymphadenopathy, and hepatosplenomegaly. Acute gastric volvulus secondary to massive splenomegaly represents an exceedingly rare yet life-threatening complication that requires urgent intervention. We reported the case of a five-year-old girl who was diagnosed with IM. Subsequently, the patient identified a 180° gastric volvulus associated with splenomegaly. Laparoscopic surgery derotation was promptly performed, with immediate resolution of symptoms. The patient experienced an uncomplicated recovery and remained asymptomatic at the 12-month follow-up. We presented a case of acute gastric volvulus as a rare complication of IM. This case underscores that in pediatric patients presenting with acute abdomen and splenomegaly, gastric volvulus should be considered alongside other complications such as splenic rupture. Prompt surgical evaluation is warranted when clinical suspicion arises.

## Introduction

Pediatric gastric volvulus represents a rare yet life-threatening clinical entity, characterized by an abnormal gastric rotation exceeding 180° and a notably lower incidence compared to adults [1]. Gastric volvulus is itself a relatively uncommon condition, with its incidence peaking after the age of 50 and adults accounting for 80-90% of reported cases [2]. Its rarity in the pediatric population further complicates timely recognition and diagnosis [3]. Etiologically, it is classified as primary or secondary. Primary gastric volvulus is defined by the inherent abnormalities of the gastric suspensory ligaments, including agenesis, elongation, or disruption. The secondary form, which constitutes approximately two-thirds of cases, arises due to associated anatomical defects such as paraesophageal hernia, diaphragmatic hernia/eventration, or phrenic nerve paralysis. Furthermore, anatomical anomalies of adjacent organs, particularly the stomach or spleen, may also predispose individuals to secondary volvulus [4]. Children often present with nonspecific symptoms that mimic common benign gastrointestinal disorders, frequently resulting in delayed diagnosis. A pathognomonic feature in acute settings is Borchardt's triad, characterized by severe epigastric pain, nonproductive retching, and failed nasogastric tube insertion. It is present in approximately 70% of cases. Delay in management risks gastric ischemia and perforation, with reported mortality rates reaching 30-50% in acute pediatric cases [5]. Owing to the absence of standardized diagnostic or therapeutic protocols, the management of pediatric gastric volvulus remains poorly characterized in the literature, often relying on anecdotal evidence.

Infectious mononucleosis (IM) is a common infectious disease among children in China, characterized by hyperplasia of the mononuclear-macrophage system. Its typical clinical manifestations include fever, pharyngitis, lymphadenopathy, hepatosplenomegaly, and an increase in atypical lymphocytes, with the primary pathogen being the Epstein-Barr virus (EBV) [6]. While IM generally has a favorable prognosis and follows a self-limiting course, its rare complications warrant careful attention. Studies have reported that IM can affect multiple systems, leading to complications such as splenic rupture (with an incidence of 0.1-0.5%) and lymphoproliferative diseases [7,8]. Notably, IM is often accompanied by significant hepatosplenomegaly. These changes not only increase the overall pathological burden on patients but, more importantly, the substantial enlargement of intra-abdominal organs alters the normal anatomical layout and spatial relationships within the abdominal cavity. This alteration can lead to changes in the tension of the gastric ligaments or compression of supporting structures, thereby providing a potential anatomical basis for the development of severe mechanical complications such as gastric volvulus [9]. Notably, the concurrent presentation of acute gastric volvulus and IM has not been systematically described. Here, we report, to our knowledge, the first documented case of simultaneous gastric volvulus in a pediatric patient with active IM.

## Case presentation

A five-year-old girl presented with a two-day history of fever, sore throat, and neck masses. Her past and family history were unremarkable. At admission, her temperature was 36℃, respiratory rate 28 times/min, pulse 106 times/min, and weight 18 kg. Physical examination revealed periorbital edema and multiple enlarged cervical lymph nodes (largest 2×1 cm), which were non-tender, mobile, and well-demarcated. Marked pharyngeal redness and bilateral grade II tonsillar enlargement with extensive whitish exudate were observed. Abdominal examination showed the spleen was felt 3 cm below the left costal edge and was firm. The remainder of the examination was non-contributory.

Laboratory tests revealed leukocytosis (white blood cell count increased with lymphocytic predominance). Hemoglobin and platelet counts were within normal limits. Slightly elevated C-reactive protein and an increased atypical lymphocyte percentage were noted. Liver function tests showed significant aspartate aminotransferase and alanine aminotransferase (Table 1). EBV nucleic acid quantification was 2.312×104 copies/mL, and EB-VCA-IgM was positive. Abdominal ultrasonography indicated hepatomegaly and splenomegaly (thickness 4 cm and length 10.7 cm; reference: thickness less than 3 cm and length less than 9 cm), both with homogeneous parenchymal echogenicity and no focal lesions.

**Table 1:** Admission laboratory values

Parameters	Patient results	Reference ranges
White blood cell count	16.74×10^9^/L	4.4-11.9×10^9^/L
Lymphocyte percentage	72.5%	33-69%
Lymphocyte count	11.53×10^9^/L	1.8-6.3×10^9^/L
Hemoglobin	125 g/L	112-149 g/L
Platelet count	225×10^9^/L	188-472×10^9^L
High-sensitivity C-reactive protein	11.35 mg/L	<5 mg/L
Atypical lymphocyte percentage	12%	0%
Alanine aminotransferase	101 IU/L	7-30 IU/L
Aspartate aminotransferase	225 IU/L	14-44 IU/L
EB-DNA	2.312×10^4^ copies/mL	Negative

The patient was admitted to the pediatric ward for supportive care after being diagnosed with IM. The treatment regimen included bed rest, hepatoprotective therapy with glutathione, and symptomatic management. Although the initial symptoms showed improvement after five days of therapy, the patient suddenly developed acute abdominal pain and vomiting on the fifth day of hospitalization. Physical examination revealed abdominal distension and tenderness without peritoneal irritation signs, accompanied by reduced bowel sounds. Despite conservative measures such as fasting and electrolyte correction, the condition did not improve, and nasogastric tube insertion was difficult during gastrointestinal decompression. Consequently, immediate imaging evaluation was performed.

**Figure 1:**
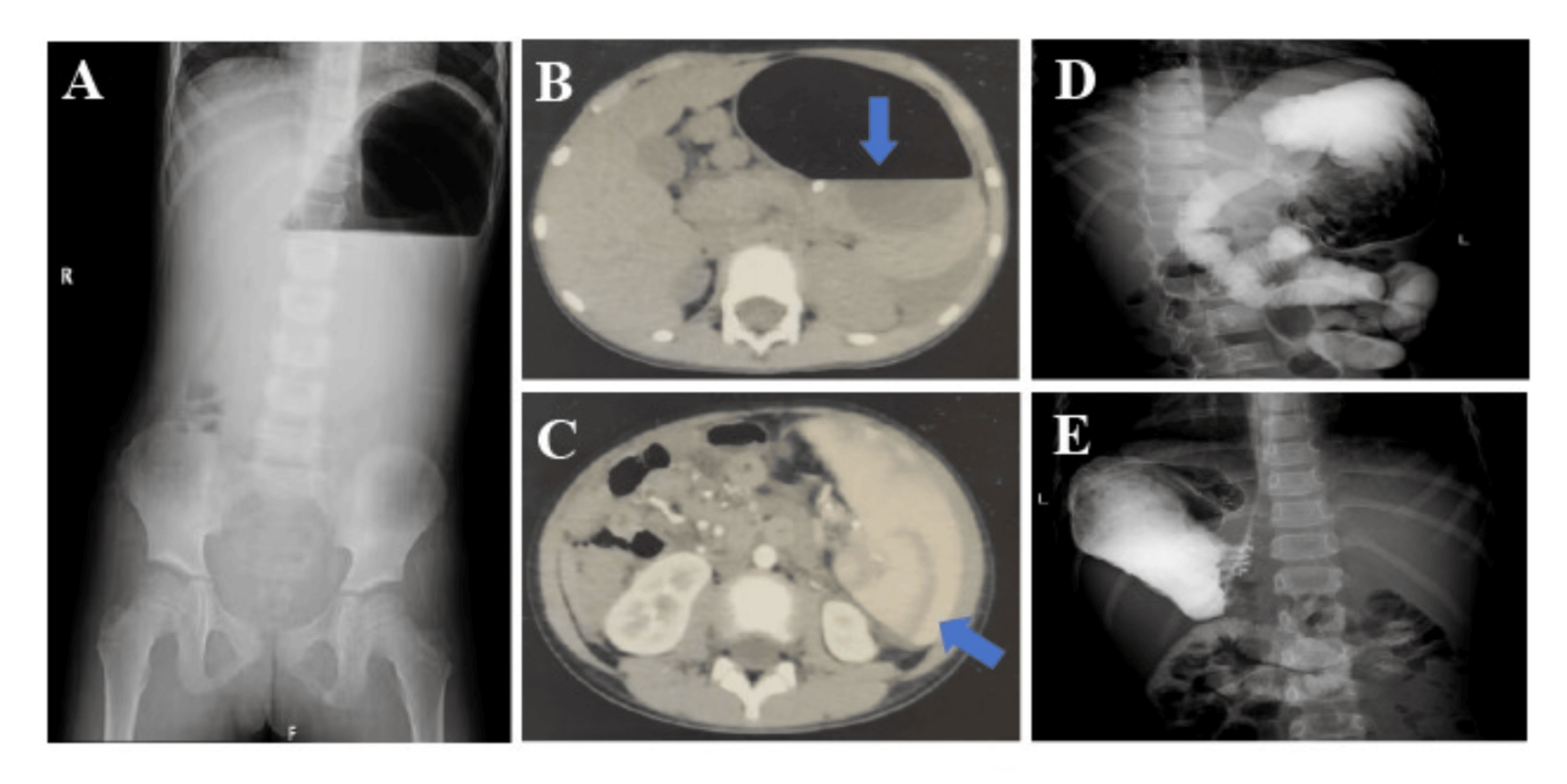
Abdominal image finding (A) Upright abdominal plain film demonstrated gastric outlet obstruction with dilatation and stasis. (B) Abdominal CT showed a distended stomach with retained contents (blue arrow). (C) The CT highlighted significant splenomegaly (blue arrow). (D-F) Upper gastrointestinal series revealed a complete 180° organoaxial gastric volvulus (the greater curvature situated above the lesser curvature). CT: computed tomography

The abdominal ultrasound examination did not reveal any abnormalities. However, due to the persistence of vomiting and abdominal pain, additional imaging studies were promptly pursued. The upright abdominal radiograph revealed features consistent with gastric outlet obstruction, including gastric dilatation and stasis (Figure 1A). These findings were confirmed on abdominal computed tomography (CT), which demonstrated a distended stomach with retained intraluminal contents (Figure 1B). The CT study further identified marked splenomegaly (length 12.46 cm, width 10 cm, thickness 9.78 cm) (Figure 1C). Upper gastrointestinal series disclosed a complete 180° organoaxial gastric volvulus and mild peri‑gastric ligament laxity, characterized by elevation and rotation of the gastric body with inversion of the normal curvature anatomy (Figure 1D-1E). Due to persistent vomiting and clinical severity, urgent laparoscopic surgery was undertaken, revealing massive splenomegaly compressing the pyloroduodenal region and a 180° clockwise gastric volvulus. Laparoscopic surgery derotation promptly relieved symptoms. Postoperative imaging demonstrated resolution. Recovery was uncomplicated, with discharge on postoperative day 5 with normal liver function. The patient remained well at the 12-month follow-up, with no recurrent symptoms and normal gastric motility and feeding pattern, confirming the safety of the gastropexy regarding long-term gastric function.

## Discussion

We reported the first pediatric case of gastric volvulus secondary to IM. After the confirmed diagnosis of IM, the patient received symptomatic supportive therapy and bed rest. Although her initial symptoms gradually improved, her condition deteriorated sharply on the fifth day of hospitalization, manifesting the classic Borchardt's triad (sudden severe epigastric pain, vomiting, and difficulty in nasogastric tube drainage). This triad is a well-established clinical hallmark for diagnosing acute gastric volvulus. Radiological assessments strongly suggested the possibility of gastric volvulus and effectively ruled out other differential diagnoses such as intussusception, hypertrophic pyloric stenosis, and intestinal obstruction, thereby prompting immediate laparoscopic intervention. Intraoperatively, a 180° clockwise gastric volvulus was confirmed, and subsequent surgical reduction promptly alleviated her symptoms.

Anatomically, the normal positioning of the stomach is maintained by several ligaments (gastrocolic, hepatogastric, gastrosplenic, gastrophrenic) that prevent abnormal rotation [9]. In this case, intraoperative observation revealed mild laxity of the gastric suspensory ligaments without any overt structural defects such as agenesis, elongation, or rupture. Coupled with the patient's lack of a prior history of gastric volvulus, these findings suggest a low likelihood of primary gastric volvulus. Additionally, we systematically excluded the common anatomical etiologies of secondary gastric volvulus: the patient exhibited no diaphragmatic abnormalities, such as paraesophageal hernia, diaphragmatic hernia/eventration, or phrenic nerve paralysis, nor were there any significant pathologies in adjacent organs. Consequently, the marked splenomegaly induced by IM emerges as the most probable and sole mechanical precipitant for this acute gastric volvulus. This combination ultimately led to gastric rotation and consequent obstructive symptoms, such as abdominal pain and vomiting. It is noteworthy that a "wandering spleen", a rare condition wherein the spleen migrates to the lower abdomen or pelvis, has been occasionally reported in association with gastric volvulus [10,11]. However, this patient did not have a wandering spleen. Although extremely rare in children, gastric volvulus must be considered in the differential diagnosis of acute or recurrent abdominal pain accompanied by vomiting. It is crucial to recognize that rotations exceeding 180° can lead to complete obstruction with risks of ischemia, incarceration, necrosis, or perforation. Without prompt treatment, the mortality rate is high; Cole and Dickinson documented a mortality rate of 65% in case reports involving children with acute gastric volvulus prior to 1950 [12].

Acute gastric volvulus presents with a broad spectrum of symptoms, complicating its timely recognition. In the absence of standardized imaging protocols, imaging choices often depend on individual clinical judgment and presentation. In pediatric cases, initial assessment typically involves abdominal radiography; findings may include a dilated gastric bubble or air-fluid levels in the upper quadrants, as observed in our patient. Furthermore, it often fails to reveal volvulus below the diaphragm. Moreover, a barium upper gastrointestinal series has historically been used for diagnosing volvulus in children, particularly for visualizing the axis of rotation and demonstrating obstruction. However, they are time-intensive and may not be suitable for acutely ill patients. Although ultrasound offers the advantages of being radiation-free and readily available, its findings are frequently subtle and operator-dependent and can be obscured by gaseous distension, contributing to a reported diagnostic accuracy of less than 25% for this condition [13]. Given these diagnostic constraints, emergent surgical exploration may be mandatory when gastric volvulus is suspected.

The case presented the first documented case of gastric volvulus in a pediatric patient with IM, thereby providing notable educational insight. It highlights the importance of considering gastric volvulus in the differential diagnosis of pediatric IM patients experiencing acute abdominal pain and vomiting. Through the continued reporting of such atypical presentations, our understanding of pediatric IM and its potential role in upper gastrointestinal obstruction will evolve, guiding further mechanistic investigation and optimization of clinical practice.

## Conclusions

Gastric volvulus is a rare yet potentially life‑threatening emergency in pediatric patients. We report a case in which acute gastric volvulus occurred as an unusual complication of IM, accompanied by splenomegaly. The obstruction was ultimately relieved through surgical reduction. This case underscores the importance of considering gastric volvulus in patients with splenomegaly who present with acute abdominal symptoms. Timely imaging studies and surgical intervention are essential. When an acute abdomen is accompanied by features such as Borchardt's triad, underlying gastric volvulus should be suspected. Therefore, unless it is included in the differential diagnosis, gastric volvulus is easily overlooked. Our experience advocates for prompt surgical assessment when clinical suspicion arises and contributes to awareness of this rare gastrointestinal complication in susceptible populations.
